# Environmental and Mechanical Performance of Green Concrete Utilizing Coarse Copper Slag Aggregate

**DOI:** 10.3390/ma19143142

**Published:** 2026-07-22

**Authors:** Sandra Guševac, Vesna Marjanović, Olivera Đokić, Aleksandar Radević, Sandra Milutinović, Jelena Đorđević, Dragana Adamović Marković

**Affiliations:** 1 Mining and Metallurgy Institute Bor, Alberta Ajnštajna 1, 19210 Bor, Serbia; vesna.marjanovic@irmbor.co.rs (V.M.); sandra.milutinovic@irmbor.co.rs (S.M.); jelena.djordjevic@irmbor.co.rs (J.Đ.); dragana.adamovic@irmbor.co.rs (D.A.M.); 2 The Highway Institute, Belgrade, Bulevar Peka Dapčevića 45 (Kumodraška 257), 11010 Belgrade, Serbia; o.djokic@highway.rs; 3 Faculty of Civil Engineering, The University of Belgrade, Bulevar kralja Aleksandra 73, 11000 Belgrade, Serbia; aradevic@grf.bg.ac.rs

**Keywords:** green concrete, coarse copper slag aggregate, solidification, EA NEN 7375

## Abstract

This study investigates the environmental potential and viability of replacing natural river aggregates (RAs) with copper slag aggregates (CSAs) in concrete production. The primary objective was to assess the structural performance of these eco-concrete mixtures and determine the optimum copper slag content for structural applications. The experimental program evaluated concrete mixtures with natural river aggregate replacement levels of 20% + 20% and 50% + 100% for the 8/16 mm and 16/32 mm fractions, respectively, using coarse copper slag aggregate (CCA). The results indicate that incorporating CCA increases concrete compressive strength, successfully meeting the requirements for strength class C25/30. The petrographic assessment indicated a shift towards an aggregate mixture, in which the dominant quartzite and a constant quartz-mineral fraction of 16.5% provide a stable structure alongside the CSA grains. However, a significant increase in water penetration depth (up to 22%) was observed, highlighting the enhanced water penetration depth of these concretes. SEM microstructural analysis attributed the improved bond between the cement matrix and CCA grains to a compact interfacial transition zone. Additionally, leaching tests confirmed that heavy metals are effectively immobilized in the cement paste for mixtures with lower replacement levels (up to 20%), thereby meeting environmental standards. The study concludes that copper slag at these controlled replacement levels represents a sustainable, high-quality alternative for construction materials in drainage infrastructure. Incremental analysis in accordance with NEN 7375 showed that the tested material behaves as an insoluble matrix, with no evidence of diffusion-controlled leaching. The cumulative leaching values obtained after 64 days of testing in accordance with NEN 7375 were significantly below the regulatory limits for all components analyzed. These findings indicate a low potential for contaminant release and favorable environmental stability of the 20% replacement mixture, though further leaching evaluation is required for maximum slag contents.

## 1. Introduction

The global construction industry is under increasing pressure due to the accelerated depletion of natural mineral resources. Concrete is widely used in building construction, bridge engineering, and road construction, as well as in the production of many prefabricated concrete elements, such as curbs and gutters [1]. Concurrently, the metallurgical industry generates large quantities of by-products, such as copper slag, which are stored in landfills [2]. In regions with intensive mining activities, the volume of copper slag in landfills has reached millions of tons, requiring urgent measures for its reuse [3]. The use of this industrial waste as a replacement for coarse aggregate in concrete production represents a reliable path toward sustainable development [4]. This approach addresses the problem of mining waste disposal while simultaneously reducing the need for the exploitation of natural resources, thereby achieving a twofold environmental contribution through the rationalization of raw material consumption [5].

Over the past decade, extensive research has evaluated the potential of copper slag as a partial replacement for fine aggregate in concrete mixtures. Numerous studies have shown that using copper slag as a replacement for river sand (0/4 mm) generally improves the workability and compressive strength of concrete, thanks to its high bulk density and low water absorption [6]. However, the application of copper slag as a replacement for coarse aggregate (e.g., fractions 8/16 mm and 16/32 mm) remains significantly less explored [7]. Some studies suggest that the glassy surface and angular grain shape of copper slag can improve the interlocking mechanism with the cement paste, further enhancing the characteristics of such concretes [8]. There is a noticeable lack of research regarding the application of copper slag aggregates in green concrete to define the relationship between high water permeability and increased mechanical strength [9]. Understanding how coarse fractions of copper slag affect the internal pore structure and heavy metal leaching behavior is essential to confirm the long-term sustainability of such materials [10].

To fill this identified gap in the literature, this research provides a comprehensive evaluation of green concrete containing coarse fractions of CCA as a replacement for natural aggregate (NA) in fractions 8/16 mm and 16/32 mm [11,12]. The experimental program is designed to assess the correlation between mechanical characteristics and environmental impact at replacement percentages ranging from 20% to 100% of CCA in concrete [13,14]. In addition to standard testing of physical and mechanical properties, the significance of the research lies in the parallel evaluation of the tested concrete characteristics in relation to its environmental feasibility [15]. Special emphasis is placed on the potential mobilization of heavy metals through rigorous leaching tests, ensuring that the concrete’s high permeability does not compromise groundwater quality. The results of this study are expected to provide practical guidelines for the recycling of mining waste and its application in construction, thereby supporting the principles of sustainable development [16].

According to previous studies [17], and the protocol of the NEN 7375 method [13] the cumulative release (E_i_) and time (t_i_) for each leaching test interval were analyzed to obtain the slope of the curve (rc_a–b_), which quantitatively characterizes the leaching mechanisms from the solidified material (as shown in Equations (1)–(8)). Therefore, rc_a–b_ values ≤ 0.35 correspond to surface wash-off or depletion as the primary mechanisms controlling the release of leaching behavior of inorganic components (ICs), while values between >0.35 and ≤0.65 indicate that the primary mechanism is diffusion-controlled release, and values >0.65 suggest that dissolution is the dominant mechanism [18].

The obtained eluate concentrations were recalculated and reported as mg/kg dry matter (d.m.), in accordance with the Serbian regulatory framework for waste characterization and classification. The results were subsequently evaluated against the relevant waste acceptance criteria for landfill disposal [19].

## 2. Materials and Methods

**Figure 1:**
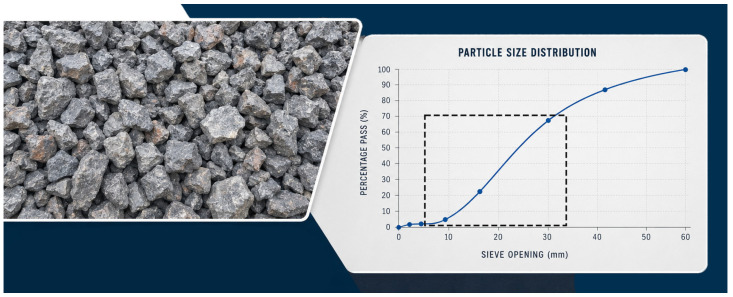
CCA disposal site at the landfill. Explanation: The dashed square highlights the presence of 70% aggregate from the 8/16 mm and 16/32 mm fractions.

The copper slag was obtained from the copper processing operations at the smelting plant in Bor, Serbia. It belongs to the category of non-hazardous industrial waste, characterized by a greenish ocher–purple color, a glassy structure, and an angular, polyhedral particle shape (Figure 1). The copper slag is formed at the landfill through rapid cooling of hot copper slag, whereby, due to the input and output requirements of the technological process, large lumps (up to 600 mm) are produced.

Based on the grain size distribution curve of the initial landfill sample, 70% of the total copper slag aggregate at the landfill consists of the 8/16 mm and 16/32 mm fractions. For this reason, and to ensure the economic feasibility of using copper slag in concrete production, the research focused on these coarse fractions (8/16 mm and 16/32 mm). The physical and mechanical properties of the CCA and natural aggregate are presented in Table 1.

As a reference natural aggregate in this research, river aggregate from the Velika Morava River deposit in the vicinity of Paraćin was used. The natural river aggregate used in this study was not crushed; it consisted of gravel with naturally rounded particles. The testing encompassed four standard aggregate fractions: 0/4 mm, 4/8 mm, 8/16 mm, and 16/32 mm.

The physical and mechanical properties of the natural aggregate (RA) and CCA are presented in Table 1. All data were taken from a previous study and are included here solely for basic characterization of the raw aggregate materials used in concrete production. These values are not intended for direct comparison between natural river aggregate and copper slag aggregate tested in different grain size classes [3]. In contrast, the chemical composition of the coarse copper slag aggregate (CCA) is shown in Table 2.

As a binder in this research, Portland cement designated as CEM II/A-L 42.5R (Moravacem, Popovac, Serbia), conforming to the European standard EN 197-1 [20], was used. The specific gravity of the used cement is 3.15 g/cm^3^. Physical and mechanical properties were determined in compliance with EN 196 [21] series standards. Its physical and mechanical properties are presented in Table 3.

The natural aggregate used in this study comes from the Velika Morava River near Paraćin (Serbia). Four standard river aggregate fractions were used, 0/4 mm (I), 4/8 mm (II), 8/16 mm (III), and 16/32 mm (IV), obtained at the separation plant “Nova separacija JUGA COOP” (Paraćin, Serbia). The particle size distribution of the aggregates was determined in accordance with EN 933-1 [22], while the simplified petrographic description and the qualitative and quantitative determination of the natural river aggregate composition were performed in accordance with EN 932-3 [23]. Based on the replacement levels of the coarse natural aggregate fractions with copper slag aggregate, the petrographic compositions of the resulting aggregate mixtures were subsequently calculated. 

The quantities of individual aggregate fractions used in the concrete mixtures are presented in Table 4. The same fraction proportions were applied in all mixtures. Based on these fraction proportions and the defined replacement levels of fractions III and IV with copper slag aggregate, the modified aggregate compositions were calculated. Fractions 0/4 mm (I) and 4/8 mm (II) remained unchanged, whereas the replacements were applied to fractions 8/16 mm (III) and 16/32 mm (IV).

The petrographic compositions of the river aggregates, as well as the calculated petrographic compositions of the aggregate mixtures containing different percentages of copper slag aggregate, are presented in Section 3.2. The partial replacement of the coarse natural aggregate fractions with copper slag aggregate altered the overall petrographic composition of the aggregate used in the concrete mixtures.

**Figure 2:**
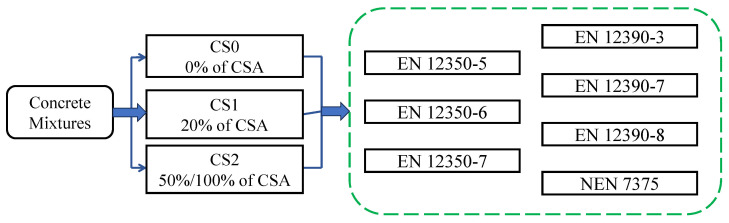
Concrete mixtures testing matrix.

The experimental program involved evaluating the physico-mechanical properties of three distinct groups of concrete mixtures, tested in compliance with the standards outlined in Figure 2.

Six aggregate compositions were initially calculated to evaluate the influence of different CCA replacement levels on the overall petrographic composition of the aggregate mixture. However, only three compositions were selected for concrete production and further testing: the natural river aggregate composition used in CS0, the 20% replacement of fractions III and IV used in CS1, and the 50% replacement of fraction III and 100% replacement of fraction IV used in CS2. The replacement of the 8/16 mm and 16/32 mm river aggregate fractions with copper slag aggregate was performed on a volume-equivalent basis. The corresponding mass quantities were then calculated based on the different particle densities of the river aggregate and the copper slag aggregate. All mixtures were engineered using commercial mix designs from a local concrete batching plant, thereby highlighting the viability of these concretes for direct industrial applications.

Immediately after mixing and before casting into the molds, the properties of the fresh concrete were tested: consistency by the flow table test (EN 12350-5 [24]), density (EN 12350-6 [25]), and air content (EN 12350-7 [26]).

Testing of hardened concrete density (EN 12390-7 [27]) and compressive strength (EN 12390-3 [28]) was conducted on cubic specimens with a side length of 150 mm. The compressive strength was evaluated at concrete ages of 1, 7, and 28 days.

The resistance to water penetration under pressure was evaluated on 28-day-old specimens in accordance with EN 12390-8 [29] at a constant water pressure of 5 bar for 72 h. While this standard is traditionally used to verify the impermeability and durability of conventional concrete, in this study it was used as a comparative indicator to assess the relative connectivity of the internal pore structure and the potential vulnerability to pressurized fluid penetration.

To investigate the microstructure and bond quality between the cement paste and the aggregate grains (both RA and CCA), a scanning electron microscopy (SEM) analysis was performed. For this analysis, small fragments of concrete specimens collected after the 28-day compressive strength testing were used. Special attention was paid to the interfacial transition zone (ITZ) between the cement matrix and both aggregate types to assess the bond’s compactness.

The diffusion test, in accordance with the standard NEN 7375 [30], is used to evaluate the long-term leaching of substances from monolithic materials, such as hardened industrial wastes, construction materials, and other stabilized wastes, into water intended for disposal, recycling, or reuse. The method is based on immersing a monolithic sample of known dimensions in deionized water at a defined liquid-to-solid phase ratio, under controlled laboratory conditions. During the total test period of 64 days, the eluate is completely removed by decantation at predefined time intervals (0.25, 1, 2.25, 4, 9, 16, 36, and 64 days) and replaced with the same volume of fresh deionized water. The obtained eluates are analyzed to determine the physicochemical parameters and concentrations of potentially mobile components, which enables the evaluation of the diffusion behavior, degree of washability, and ecological acceptability of the tested material [31,32].

To perform the diffusion test NEN 7375 [30], a laboratory container with a lid (a polypropylene container with a volume of 2–10 L) and deionized water with an electrical conductivity of less than 10 µS/cm were used. The monolithic sample was placed in a laboratory container, after which deionized water was added at the predefined liquid-to-solid phase ratio (L/A). The container was hermetically sealed and stored at room temperature (20 ± 5 °C) throughout the experiment to ensure controlled test conditions and reproducibility of the results. pH values were determined using a calibrated pH meter, and electrical conductivity was determined using a calibrated conductometer. At the same time, metal concentrations were analyzed by ICP-MS using an Agilent 7900 ICP-MS instrument (Agilent Technologies, Santa Clara, CA, USA; manufactured in Japan). Ion chromatography (940 Professional IC Vario MagIC (Metrohm, Herisau, Switzerland)) was used to determine anions, including chlorides, sulfates, and fluorides. The eluate was changed at defined time intervals: after 0.25, 1, 2.25, 4, 9, 16, 36, and 64 days. At the end of each interval, the water from the previous contact period was decanted and taken for analysis. A fresh volume of deionized water equal in volume was added to the sample. The collected eluate samples were filtered through a 0.45 µm membrane filter and then stored under appropriate conditions until chemical analysis.

After analyzing the eluate using ICP-MS in accordance with EN ISO 11885 [33], the concentrations of the tested elements were determined in mg/L. In accordance with the requirements of the standard NEN 7375, the results were recalculated and expressed as the amount of emitted substance per unit dry mass of the sample area (mg/m^2^ dm), enabling comparison of results across different monolithic samples regardless of their dimensions. The total amount of the eluted substance was determined by summing the emissions across all time intervals, while the diffusion profile was monitored throughout the test period.

The geometric surface area of the specimens was determined using the paper method in accordance with Section 7.2.3 of NEN 7375. The outline of the irregular surface was traced onto paper of known weights in grams, and subsequently cut out. The mass of the paper template was measured, and the corresponding surface area was calculated from the ratio of the template mass to the paper weights in grams. This procedure enabled the accurate determination of the exposed leaching surface of specimens with irregular geometry.

According to the requirements of the NEN 7375 standard, Criteria 1 and 2 were checked to assess whether the samples were soluble.

If the pH and conductivity of the measured eluates indicate matrix dissolution during the test, the following calculations must be performed. An assessment must be made to determine whether Criteria 1 and 2 are satisfied. If neither criterion is satisfied, the components Ca, F^−^, SO_4_^2−^, and Cl^−^ must be determined to verify whether dissolution has occurred.

Criterion 1 was checked according to the following equation:

(1) S7−8>1.5·VpV+10^pH7−8−11.78+10^(2.5−pH7−8)

where

V—volume of the leaching fluid, (L);
V_p_—volume of the test piece, (L);
S_7–8_—the average value of the measured conductivities in periods 5 and 6 (µg/cm);
pH_7–8_—the average pH value of the measured in periods 7 and 8.

The pH value for the fifth between 7 and 8 pH_7–8_ for sample CS0 is 9.62, and for sample CS1, 20% of CS 8.62. According to the calculation, Criterion 1 was satisfied. This means that the eluate must be analyzed for Ca, F^−^, SO_4_^2−^, and Cl^−^ to demonstrate dissolution of the material and to continue to Criterion 2.

Criterion 2 requires a check if

(2) $$S_{7-8}>2\times S_{5-6}$$

where

$S_{7-8}$—the average value of the measured conductivities in periods 7 and 8 (µg/cm);
$S_{5-6}$—the average value of the measured conductivities in periods 5 and 6 (µg/cm).

Conductivity for sample CS0 in the period S_7–8_ is 96.5 and for the period S_5–6_ is 149.5. Conductivity for sample CS1, 20% of CS in the period S_7–8_, is 101.0, and for the period S_7–8_ is 101.5. For both samples, Criterion 2 is not met, which means that the samples are insoluble, and it is not necessary to perform analysis for Ca, F^−^, SO_4_^2−^, and Cl^−^.

Calculations were performed in accordance with NEN 7375 to evaluate the leaching behavior of the investigated components. The assessment included determination of the leached amount in each eluate fraction, calculation of cumulative release, identification of the dominant leaching mechanism, and evaluation of cumulative leaching per unit area. Furthermore, surface wash-off and diffusion-controlled release were assessed, and the upper leaching limit was determined for constituents for which diffusion-controlled leaching could not be established.

The leaching of each investigated component in an individual eluate fraction was determined separately from the eluate concentration and the corresponding eluate volume, according to the procedure specified in NEN 7375, point 8.1:

(3) $$E_{i}^{*}=\frac{c_{i}\cdot V}{f\cdot A}$$

where

$E_{i}^{*}$—measured leaching of a component in fraction i, (mg/m^2^);
$c_{i}$—concentration of the component in fraction i, (µg/L);
V—volume of the eluate, (L);
A—surface area of the test peace, (m^2^);
F—conversion factor, 1000 µg/mg.

The concentration $c_{i}$ specified in the formula is then the concentration originally present in the eluate. Supposing that the concentration of a component in a specified fraction is below the lowest limit of analytical determination, two calculations must be carried out for the component. The upper limit of $E_{i}^{*}$ is calculated by equating $c_{i}$ in the formula with the lowest limit determination; the lower limit of $E_{i}^{*}$ is calculated by setting $c_{i}$ in the formula to 0.

For each component to be analyzed, calculate separately the measured cumulative leaching $\varepsilon_{n}^{*}$ in each of the periods n = 1 to N, where n = 1 lasts from the start of the test to the first replenishment time. Period n = 2 is from the start of the test to the second replenishment time (comprises fraction 1 + 2), etc. (NEN 7375, point 8.2.1). Carry out these calculations as

(4) $$\varepsilon_{n}^{*}=\sum_{i=1}^{n}E_{i}^{*},\mathrm{for}\;n=1\mathrm{to}N.$$

where

$\varepsilon_{n}^{*}$—measured cumulative leaching of a component for period n comprising fraction i = 1 to N, in mg/m^2^;
$E_{i}^{*}$—measured leaching of the component in fraction n *i* in mg/m^2^;
N—number of periods equal to the number of specified replenishment times (N = 8).

For each component to be analyzed, calculate separately the derived cumulative leaching $\varepsilon_{n}$ in each of the periods n = 1 to N, where a period n lasts from the start of the test to the nth replenishment time (comprises fraction i = 1 to N) (NEN 7375, point 8.2.2).

Carry out this calculation as follows:

(5) $$\varepsilon_{n}=(E_{i}^{*}\cdot \sqrt{t_{i}})/(\sqrt{t_{i}}-\sqrt{t_{i-1}})$$

where

$\varepsilon_{n}$—derived cumulative leaching of a component for period n comprising fraction i = 1 to N, in mg/m^2^;
$E_{i}^{*}$—measured leaching of the component in fraction *i* in mg/m^2^;
$t_{i}$—replenishment time of fraction i, i.e., time at end of fraction *i*, in s;
$t_{i-1}$—replenishment time of fraction i − 1, i.e., time at start of fraction *i*, in s.

For each component under study, an incremental analysis was performed by component as follows (NEN 7375, point 8.3.2):

Step 1: For each increment a–b, the concentration factor CF_a–b_ was determined:

(6) $${CF}_{a-b}=\frac{mean\;concetration\;in\;the\;increment}{lowest\;limit\;of\;determination}$$

If in all fractions in the increment a–b the measured concentrations for the component under study were greater than the lower limit of determination for that component, and if CF_a–b_ = 1.5, then Step 2 was continued. If this is not the case, then the leaching mechanism for this component in a particular increment cannot be determined.

Step 2: Using linear regression of the relation log ε_n_ − log t (with i = n), the slope r_c_ and the corresponding standard deviation sd_rc_ calculated from the regression analysis were determined for each increment. For each component to be studied, an incremental analysis was undertaken per component as follows:

(7) $$r_{c(a-b)}=({lgE}_{a}-{lgE}_{b})/({\mathrm{lg}t}_{a}-{\mathrm{lg}t}_{b})$$

According to point 8.6.1, the upper leaching limit was determined by the following formula (NEN 7375, point 8.6.1):

(8) $$\varepsilon_{T}=5\cdot \varepsilon_{1-8}^{*}\cdot \sqrt[{2}]{\frac{T}{64}}$$

where

$\varepsilon_{T}$—upper leaching limit (mg/m^2^);
$\varepsilon_{1-8}^{*}$—measured cumulative leaching for a component per unit surface area over 64 days (mg/m^2^);
T—period of test duration (days).

## 3. Results

### 3.1. Concrete Composition and Characteristics of Fresh Concrete

**Figure 3:**
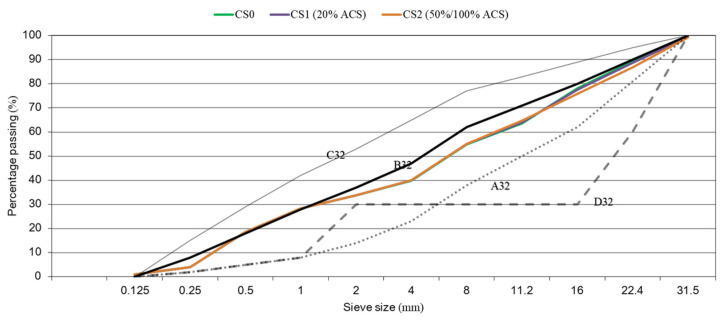
Particle size distribution of the tested mixtures: A32—the lower bound of the optimal area; B32—average curve; C32—upper limit; D32—curve for discontinuous granulometric composition.

To investigate the influence of CCA on the properties of fresh and hardened concrete, three mixtures were designed with varying replacement percentages of natural aggregate with copper slag aggregate. The detailed aggregate grading of all tested mixtures is systematized and presented in Figure 3.

This composition, with a higher proportion of fine aggregate, was deliberately adopted to achieve a very compact, dense structural skeleton. Such a framework is necessary for the development of environmentally efficient green concrete with higher mechanical properties, rather than a porous matrix with an open structure. The control mixture was designed to meet the requirements of strength class C25/30, which is the most widely used concrete class in the construction industry’s total production [12]. During the mix design process, the mass proportions of cement, water, and fine fractions of the river aggregate were kept constant. In contrast, a partial replacement with copper slag aggregate was performed for the remaining fractions (8/16 mm and 16/32 mm), as shown in Table 5. Due to the higher specific gravity of the copper slag aggregate compared to the natural river aggregate, this replacement was conducted on a volume-equivalent basis and subsequently converted into mass proportions. This methodological approach enables a clear evaluation of CCA’s influence on changes in the properties of fresh and hardened concrete.

The test results of the fresh concrete properties (consistency, density, and air content) are presented in Table 6. Analysis of the results indicates that incorporating CCA affects workability nonlinearly. At a moderate replacement level (CS1), the concrete consistency remains stable, exhibiting a negligible variation in slump (from 20 cm to 21 cm) and a minor reduction in flow. However, a further increase in CCA content (CS2) directly reduces workability, causing the concrete mix to become significantly stiffer, with the slump dropping to 13 cm and the flow to 32 cm. This behavior at higher replacement levels can be attributed to the specific angular polyhedral grain morphology and rough surface texture of the copper slag aggregate, which increase internal friction between particles and require more cement paste for particle lubrication. In contrast, mixes with higher CCA content showed a trend toward increasing fresh concrete bulk density (up to 5.40% for CS2) and decreasing entrained air content, a direct consequence of the higher specific gravity of the copper slag.

### 3.2. Petrographic Analysis of River Aggregate and Its Replacement by Copper Slag Aggregate

It should be noted that while the main focus of the investigation (as detailed in Section 2) is placed on the three selected optimal mixtures, a broader matrix of six preliminary mixtures was initially evaluated during the screening phase. To provide a comprehensive overview of the material behavior, the results for all six preliminary mixtures are displayed in this section. Based on these initial calculations, the three specific mixtures described in the methodology were selected for further comprehensive analysis.

The examined natural aggregate mainly consists of fragments of metamorphic (44.7%) rocks. Fragments of igneous (21.3%) and sedimentary rocks (17.6%) are less represented, as well as grains of minerals (16.5%) (Table 7, a). Metamorphic rock fragments are dominated by quartzite, with subordinate gneiss and minor schist. In the igneous rock group, peridotite is the most abundant component, whereas gabbro-diabase and dacite-andesite occur in lower and relatively similar proportions. Sedimentary rock fragments are mainly sandstone, with small amounts of limestone, chert, and conglomerate. The mineral grains are dominated by quartz, while feldspars, ferromagnesian minerals, serpentine, and muscovite are present only in minor amounts.

In the coarse aggregate, spherical and oblong grains are most prevalent; they are mostly incompletely rounded, with smooth and finely rough surfaces.

With the addition of copper slag in mixtures b–f, the petrographic composition changes progressively due to the partial replacement of natural aggregate particles. The content of copper slag increases from 9.0% in mixture b to 35.0% in mixture d, while mixtures e and f contain 16.5% and 32.5% copper slag, respectively. Consequently, the proportions of all natural rock fragments decrease, whereas the mineral fraction remains constant at 16.5%. The decrease is most pronounced for quartzite, which remains the dominant natural rock component in all mixtures, but its content decreases from 32.9% in the standard aggregate to 20.9–29.9% in the mixtures with copper slag. Similar decreasing trends are observed for gneiss, sandstone, peridotite, gabbro-diabase, and dacite-andesite. Sedimentary and igneous rock fragments become less represented with increasing copper slag content, while schist, chert, and conglomerate remain minor components.

Overall, the mixtures retain the basic petrographic character of the natural river aggregate, but show a gradual shift towards a composite aggregate composition containing an increasing proportion of copper slag.

**Figure 4:**
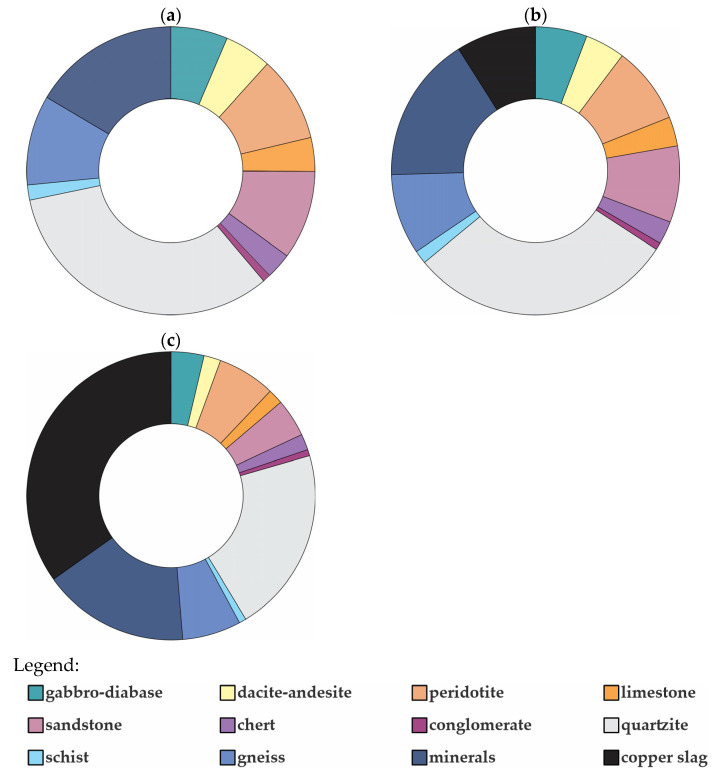
Petrographic composition of doughnuts consists of river aggregate and different percentages of copper slag aggregate: (**a**) standard river aggregate; (**b**) added 20 III and 20 IV; (**c**) added 50 III and 100 IV.

To provide a clearer visual comparison of the petrographic composition, the data from Table 7 are presented as doughnut charts in Figure 4. The diagrams show the relative proportions of the main rock groups, mineral grains, and copper slag in the standard river aggregate (Figure 4a) and in mixtures containing different percentages of copper slag aggregate (Figure 4b,c). This graphical presentation makes it easier to observe the progressive replacement of natural aggregate components with copper slag.

### 3.3. Physical and Mechanical Characteristics of Hardened Concrete

The compressive strength test results indicate that the mixtures containing copper slag aggregate (CCA) achieve higher compressive strength than the control concrete (CS0) at all ages. Specifically, at 28 days, mixtures CS1 (20% III + 20% IV) and CS2 (50% III + 100% IV) achieved strength increases of approximately 10.2% and 16.5%, respectively, relative to the control CS0 mixture.

An increasing trend was also observed in hardened concrete density, with mean 28-day increases of approximately 1.12% for CS1 and 5.84% for CS2, relative to the control mixture. On the other hand, the results presented in Table 8 indicate an increase in water penetration depth in the CCA-containing mixtures. The water penetration depth of CS1 and CS2 concretes was approximately 12.05% and 22.9% higher, respectively, than that of the control mixture, confirming their open porosity and greater penetration capacity.

The mechanical and physical properties of the hardened concrete mixtures, including individual measurements for the three distinct specimens per group and their corresponding standard deviations, are presented in Table 8. As expected, incorporating coarse copper slag aggregate significantly increased the bulk density of the hardened concrete. Specifically, the CS2 mixture exhibited a mean 28-day hardened density approximately 5.84% higher than that of the control concrete (CS0), which is directly attributable to the higher specific gravity of the slag (3.40 g/cm^3^) relative to the natural river aggregate (2.66 g/cm^3^).

### 3.4. Mineralogical Analysis of Concrete with Copper Slag Aggregates

**Figure 5:**
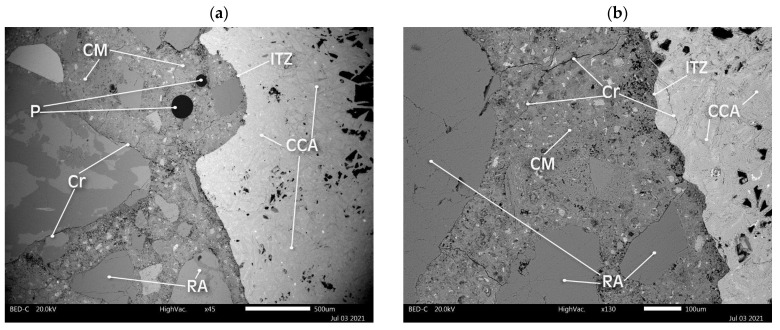
SEM micrographs of the concrete matrix with copper slag aggregate: (**a**) general overview of the microstructure and phase distribution at 45× magnification; (**b**) detailed view of the microstructural network and microcracks at 130× magnification: CCA—coarse copper slag aggregate; CM—cement matrix; ITZ—interfacial transition zone; RA—river aggregate; P—air void (pore); Cr—microcrack.

The microstructure of the concrete composite incorporating copper slag aggregate is displayed in Figure 5. At a lower magnification (Figure 5a), a distinct contrast between the phases is observable; due to its high density and iron-rich composition, the copper slag aggregate (CSA) appears as a bright, angular phase well-embedded within the matrix.

The interfacial transition zone (ITZ) between the CSA and the cement matrix (CM) visually indicates a high degree of continuity and relative compactness within the observed areas, with no significant micro-gaps or heavy accumulation of porous hydration products.

A closer inspection at a higher magnification (Figure 5b) reveals the finer details of the microstructural network. While the ITZ at the slag interface remains stable and tightly bonded, a network of fine microcracks (Cr) is visible propagating through the cement matrix and around the boundaries of the natural river aggregate (RA). These cracks are characteristic of drying-shrinkage stresses or micro-stress relief that occur during sample preparation under high vacuum.

Concrete shrinkage stresses or micro-stress relief occur during sample preparation under high vacuum. Nevertheless, the integrity of the slag–paste boundary remains stable in the analyzed sections, suggesting that the copper slag aggregate develops a strong mechanical bond with the surrounding hydration products.

### 3.5. Sample Testing Results Obtained by the Standard Method NEN 7375

The data and results obtained during testing by the standard method NEN 7375, point 7.3.2, for samples of solidified with the marks CS0 and CS1, 20% of CS are shown in Table 9 and Table 10. The paper method was used to determine the area of test samples that had irregular surfaces. The coverage factor represents the relationship between the volume of the leaching fluid and the volume of the test piece.

Calculate the weight loss m_v_ (g/m^2^) of material that has fallen off the test piece during the test in two phases: the weight loss m_va_ in stages 1 to 2 of the test and the weight loss m_vb_ in stages 2 to 8. The weight loss m_va_ in stages 1 to 2 for CS0 was 0.6051 (g/m^2^), and m_vb_ in stages 2 to 8 was 1.2182 (g/m^2^). The weight loss m_va_ in stages 1 to 2 for CS1, 20% of CS was 1.047 (g/m^2^), and m_vb_ in stages 2 to 8 was 1.595 (g/m^2^). A relatively large weight loss m_vb_ compared with m_va_ indicates the long-term integrity of the material. These mass losses can be regarded as insignificant.

**Figure 6:**
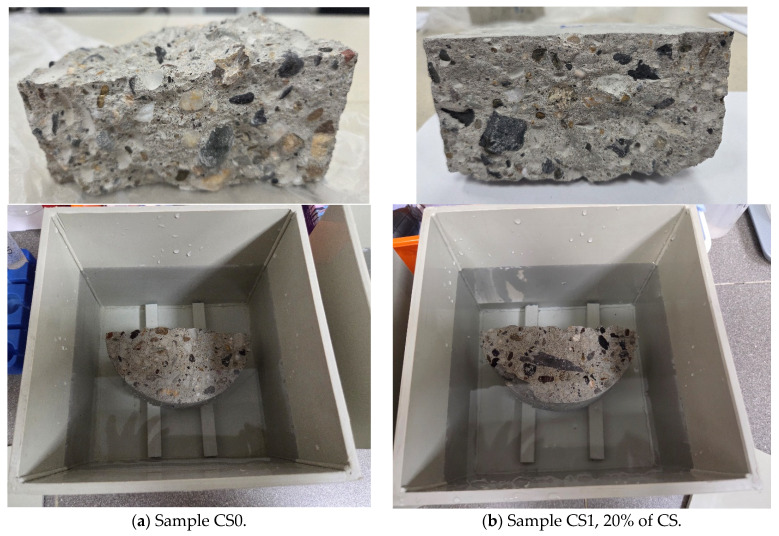
Appearance of the samples, and samples during the testing.

Figure 6 shows the appearance of the tested samples (a, b) and the tested samples during the testing using the NEN method. Due to the limited availability of the test specimens at that stage of the investigation, the leaching test was performed exclusively on the CS1 mixture.

Incremental analysis refers to estimating the amount of a constituent released in each fraction of the eluate collected during the NEN 7375 diffusion test. The obtained incremental leaching data are used to estimate the temporal pattern of release and to identify the dominant leaching mechanism governing the release of the test constituents, and are presented in Table 11. The calculations shown in Table 11, Table 12, Table 13, Table 14 and Table 15 were performed using Equations (3)–(8).

For components which ${CF}_{a-b}$ = 1.5 or higher, further analysis in Step 2 was conducted (Ca, F^−^, Cl^−^, and SO_4_^2−^) for sample CS0, and (Se, Ca, F^−^, Cl^−^, and SO_4_^2−^) for sample CS1, 20% of CS. Step 2 involves determining the concentration factor, slope, and standard deviation using incremental analysis (NEN 7375, point 8.3.2), as shown in Table 12 and Table 13.

By applying linear regression, the values for the concentration factor, the slope of the curve and the standard deviation for individual increments were obtained. In accordance with point 8.3.3 of the standard, the solubility of the matrices was determined. The results obtained are shown in Table 14. The slope of the curve for increments 5 to 8 was calculated only for parameters whose CF 5–8 ˃ 3.0. The conclusion reached by the incremental analysis is that the matrix is insoluble, which is in accordance with the conclusion according to Criterion 2, point 7.4 of the standard NEN 7375. According to point 8.3.4 of the standard NEN 7375, which defines the leaching mechanism during testing, and according to the following standard conditions, CF_a–b_ > 1.5, 0.35 < rc ≤ 0.65 sd_rc_ ≤ 0.5, as well as according to incremental analysis and Criterion 2, it follows that the matrix is insoluble.

### 3.6. Determination of Matrices’ Solubility (NEN 7375, Point 8.3.3)

Values for the concentration factor, the slope of the curve, and the standard deviation for individual increments were obtained using linear regression. According to point 8.3.3, the solubility of the matrix was determined using Criterion 3.

The obtained results are shown in Table 14. The slope of the curves for increments 5 to 8 was calculated only for components for which CF 5–8 > 3.0, which is in accordance with Criterion 3.

The conclusion reached by incremental analysis is that the matrix is insoluble. This is in accordance with the conclusion before Criterion 2, point 7.4. In accordance with point 8.3.4 of the standard, which defines the leaching mechanism during the test, and according to the following specified conditions, CF_a–b_ > 1.5, 0.35 < rc ≤ 0.65, sdrc ≤ 0.5, as well as the incremental analysis and Criterion 2, the material is insoluble.

In this case, the upper leaching limit is determined in accordance with point 8.6 of the standard—determination of the upper leaching limit (NEN 7375, point 8.6).

## 4. Discussion

The observed increase in both fresh and hardened density is directly proportional to the higher specific gravity of the copper slag aggregate compared to the river aggregate. However, the transition to a stiffer consistency with an increasing CCA content can be attributed to the morphology and rough surface texture of the copper slag aggregate particles. Unlike the rounded river gravel, the polyhedral shape of the CCA increases the internal friction between particles, thereby requiring more cement matrix to achieve the same level of workability relative to the control concrete mixture.

The observed increase in water penetration depth in CCA-containing samples should be carefully evaluated as an indicator of minor alterations in the concrete micro-porosity. In green concrete, reducing liquid penetration is desirable for durability. Experimental results demonstrate that using copper slag aggregate simultaneously enhances compressive strength and increases water penetration depth in CS1 and CS2 concrete. Typically, in concrete mixtures, an increase in porosity and permeability reduces mechanical strength; however, the improved performance of concrete mixtures containing CCA can be attributed to the high hardness of the copper slag aggregate particles, which have a more resilient mineral structure than that of natural river aggregate. The angular and polyhedral morphology of the copper slag aggregate enables superior mechanical interlocking within the cement matrix, contributing to a more stable internal structure that can withstand higher compressive loads, even with a more open pore structure. The increased water penetration depth (up to 22% for CS2) suggests that CCA slightly modifies the pore structure, creating minor macro-porosity that affects water penetration under pressure, without compromising the overall mechanical strength and internal stability of the concrete. It should be noted that frost resistance was not investigated in this study; therefore, although the tested mixtures achieved satisfactory compressive strength and environmental performance, their use in freezing- and thawing-exposed conditions requires additional testing. Consequently, future research should evaluate frost resistance to clearly define the boundary conditions for the practical application of copper slag aggregates in water-permeable concrete systems.

The observed increase in compressive strength of both CCA-containing mixtures (CS1 and CS2) compared to the control concrete (CS0) can be attributed to favorable petrographic and mechanical synergy among the aggregate components. As shown in Table 7 and Figure 4, even with the progressive introduction of copper slag, a substantial proportion of quartzite was retained in the aggregate skeleton. Considering that natural quartzite exhibits exceptionally high compressive strength, typically reported around 280–390 MPa [34], its combination with the high hardness and angular, polyhedral morphology of the copper slag aggregate grains effectively optimized the load-bearing matrix. This structural density allowed the CS2 mixture, which features the highest replacement level, to achieve a peak 28-day compressive strength of 42.3 MPa (a 16.50% increment relative to CS0). The analysis of the tested concretes indicates that the third mixture (CS2) achieved a higher strength increment (approximately 16.50%) than the second mixture (CS1, approximately 10.20%), demonstrating that the higher content of coarse copper slag aggregate further enhanced the mechanical performance. This continuous increase in mechanical performance, even at the highest replacement level (CS2), indicates that the cement matrix provided sufficient coverage and a high-quality bond around the angular CCA particles. The specific morphology of the coarse copper slag did not mitigate the structural integrity; instead, its high hardness and mechanical interlocking enhanced the overall load-bearing capacity. On the other hand, the recorded increase in water penetration depth—ranging from an 8.97% increase in the second group (CS1) to a 15.38% increase in the third group (CS2) relative to their initial stages—confirms that the higher concentration of rough, polyhedral CCA grains alters the internal pore network by creating a more interconnected macro-porosity. These findings demonstrate that while the internal pore structure becomes more open and permeable to pressurized fluid penetration, the matrix avoids any structural degradation or strength loss, achieving an optimal balance between mechanical robustness and hydraulic conductivity required for drainage infrastructure.

The water permeability of the evaluated concrete mixtures (with leaching testing focusing on CS0 and CS1) was further analyzed through microstructural analysis and an environmental sustainability assessment. The angular morphology of the coarse copper slag aggregate (CCA) directly influences the tortuosity of the channels within the concrete matrix. Although these channels facilitate high permeability, SEM analysis results confirm that the cement paste lining the channel walls is exceptionally dense, thereby preventing concrete degradation despite continuous water flow. The SEM micrograph demonstrates a high degree of compatibility between the cement matrix and the surfaces of the copper slag aggregate particles, attributed to fewer microcracks at the cement–aggregate interface. This compact bond is of paramount importance for the heavy metal leaching process. Although the mixtures exhibit an increased water penetration depth, the leaching test results prove that hazardous elements, such as Cu, Pb, and As, remain safely encapsulated within the hardened cement matrix. The alkaline environment of the cement promotes the precipitation of heavy metals as insoluble hydroxides. In contrast, the compact bond in the cement matrix–CCA interfacial transition zone serves as a physical barrier. This synergy between the physical barrier and chemical stabilization ensures that the increased concrete permeability does not lead to environmental contamination, thereby qualifying the water-permeable concrete containing CCA as an environmentally sustainable material for construction applications.

This correlation between the enhanced mechanical properties of the concrete mixtures and the increased water penetration depth classifies the water-permeable concrete containing CCA as a sustainable material for use in construction infrastructure. Replacing natural aggregate with copper slag at 20% to 100% contributes to resource conservation and the sustainable use of industrial waste. Since this concrete is intended for load-bearing structures, its environmental safety and the ability to bind heavy metals within the cement matrix are of key importance. Although the environmental behavior of the mixture with the maximum slag content (CS2) still needs to be tested in future phases, the results of the diffusion test according to the NEN 7375 standard confirm that with the optimal mixture with 20% replacement (CS1), there is no risk of leaching even in constant contact with water. Therefore, the CS1 mixture can be classified as a completely safe and environmentally friendly construction product. This nexus between the robust mechanical performance of the concrete and its environmental sustainability demonstrates that industrial waste can be successfully utilized to produce high-performance, eco-friendly construction materials.

The results of the incremental analysis carried out in accordance with the NEN 7375 standard showed that the concentrations in the eluates of most of the elements analyzed (Sb, As, Cu, Hg, Cd, Mo, Ni, and Pb) were below the detection limit. In contrast, the concentration factors (CF_a–b_) for all the analyzed increments are approximately equal to the value of 1. These results indicate that during the test, there were no significant changes in eluate concentrations, suggesting that leaching of these elements is very low Therefore, it was not possible to identify the dominant leaching mechanism for the mentioned components, which is marked as “n.a.” in the results (not applicable).

In the analyzed elements (Ca, F^−^, Cl^−^ and SO_4_^2−^ in both samples, as well as Se in sample CS1, 20% of CS), the concentration factors were greater than 1.5, which, according to NEN 7375, required additional analysis to determine the possible leaching mechanism. The obtained values of the concentration factors were particularly high for calcium and chlorides, indicating that these components are present at higher concentrations in the eluate than the other analyzed parameters. Such behavior is expected in mineral matrices, as calcium and soluble salts are often among the most mobile components of the system.

Analysis of the slope of regression lines (r_c_) showed that negative values were obtained for all examined components. A negative slope indicates a decrease in eluate concentration over time, i.e., a decrease in leaching intensity as the test progresses. This trend is characteristic of the initial washing of readily available components from the sample surface, after which the system stabilizes, and a significant reduction in the further release of substances occurs.

For most of the increments analyzed, the r_c_ values ranged from approximately −0.01 to −0.59. None of the values obtained met the criterion r_c_ > 0.8, which, according to NEN 7375, indicates dissolution of the matrix as the dominant mechanism of substance release. Based on this, it can be concluded that the examined material does not show behavior characteristic of soluble matrices.

Also, the conditions for diffusion-controlled leaching were not met. According to the standard, the diffusion mechanism requires that the concentration factor be greater than 1.5, that the slope of the regression line be between 0.35 and 0.65, and that the standard deviation of the slope be less than or equal to 0.5. The obtained r_c_ values were significantly lower than the specified interval and, in most cases, negative. This indicates that transport of the investigated components through the matrix is not governed by diffusion. The results indicate that the dominant process during the test is surface wash-off and partial depletion of readily soluble components from the material’s surface layers. After removing the initially available amounts of soluble compounds, the matrix exhibits high stability and resistance to further leaching.

Based on the criteria set out in NEN 7375, the material is classified as an insoluble matrix. Such a classification indicates that the material’s structure is stable enough to prevent significant dissolution and migration of contaminants during long-term contact with water. The test results after 64 days further confirm the material’s stability. The leaching values for all analyzed heavy metals and anions are many times lower than the reference values prescribed by the Serbian Rulebook on Categories, Testing, and Classification of Waste. It is particularly significant that the concentrations of potentially problematic elements, such as arsenic, cadmium, mercury, lead, and chromium, are several orders of magnitude below the prescribed limit values.

By comparing the results for both samples, it can be concluded that there are no sig-nificant differences in their behavior during leaching. Sample CS1, 20% of CS shows slightly higher values for selenium and sulfates, while sample CS0 is characterized by shows slightly higher values for selenium and sulfates, while sample CS0 shows slightly higher values for calcium and chlorides. However, the noted differences have noare not of practical significance for environmental impact assessment, as all measured values for these samples are well below the prescribed limits. The leaching behavior of the high-est-replacement mixture (CS2) remains to be verified in future steps. Overall, the results of the NEN 7375 test indicate that the investigated CS1 matrix exhibits high chemical stability, very low potential for contaminant release, and good resistance to long-term water exposure. Such behavior indicates efficient immobilization of the analyzed elements in the material matrix and a low risk of their transfer to the environment during exploitation or disposal.

The obtained diffusion-controlled leaching behavior indicates that the environmental performance of the tested monolithic waste can be evaluated within the framework of the Serbian Rulebook on Categories, Testing and Classification of Waste (Official Gazette of the Republic of Serbia No. 56/2010, 93/2019, 39/2021, 65/2024), Annex 8, Part 2. Disposal of non-reactive hazardous materials requires eluate characterization and compliance with landfill acceptance criteria. The national waste classification framework in the Republic of Serbia has been developed in accordance with the harmonization process with the European Union environmental acquis, adopting a waste catalog structure equivalent to the European List of Waste and applying classification principles comparable to those established by Directive 2008/98/EC on waste. In this context, the assessment of waste acceptance for landfill disposal was based on leaching data from the NEN 7375 diffusion test, and the results were interpreted in accordance with the principles and acceptance criteria set out in Council Decision 2003/33/EC. Therefore, NEN 7375 is a suitable, scientifically recognized tool for supporting waste characterization, classification, and environmental assessment of monolithic waste intended for landfill disposal [21,22].

It should be emphasized that although the 20% replacement mixture (CS1) demonstrated excellent environmental safety and compliance with the NEN 7375 standard, the mixture with the maximum copper slag content (CS2) was not subjected to diffusion-leaching protocols. Consequently, the high level of environmental stabilization reported in this section is experimentally verified strictly for replacement levels up to 20%.

## 5. Conclusions

- The replacement of natural aggregate with CCA leads to an increase in both fresh (up to 5.40% for CS2) and hardened density (5.85% for CS2), primarily due to the high specific gravity of the CCA particles. Furthermore, the fresh concrete consistency remains stable at a moderate replacement level (CS1), whereas higher replacement contents (CS2) lead to a significantly stiffer consistency due to the angular morphology and rough texture of the slag grains.
- All mixtures containing CCA replacement met the requirements for strength class C25/30. The highest strength increase of 16.50% was achieved at a replacement level of CS2, proving that the copper slag aggregate particles exert a favorable effect on the load-bearing structure of the concrete.
- The water penetration depth increased (8.97%) in the mixtures with a 20% CCA replacement level, confirming that the morphology of the copper slag aggregate promotes the formation of interconnected pores, which is crucial for applications in construction infrastructure.
- SEM analysis confirmed a compact bond between the cement matrix and the CCA, indicating excellent mechanical interlocking and chemical compatibility.
- The test results prove that the coarse fractions of copper slag aggregate serve as an excellent alternative to natural aggregate, supporting the utilization of industrial waste in construction infrastructure.
- Among the investigated formulations, the CS1 mixture featuring a 20% CCA replacement level exhibited a highly favorable balance, yielding a significant 28-day compressive strength increment alongside an enhanced water penetration depth relative to the control concrete. This demonstrates that a moderate substitution of coarse natural aggregate with CCA achieves the optimal balance between mechanical strength and permeability. The results of the incremental analysis performed in accordance with NEN 7375 showed that the diffusion-controlled leaching criteria were not met for any of the investigated components. The slopes of the regression lines (rc) for all analyzed parameters were significantly lower than the threshold value of rc > 0.8 prescribed for soluble matrices, indicating that the tested material behaves as an insoluble matrix.
- The objective of the NEN 7375 test was to assess the environmental performance of the mixtures identified as the most suitable for practical application based on the overall evaluation of their mechanical and physical properties. Although CS2 contained the highest proportion of copper slag, it was not selected because it was not identified as one of the candidate formulations for further application.
- In accordance with the requirements of NEN 7375, the assessment of the upper limit of leaching is therefore applicable to this type of material.
- The leaching test results obtained after 64 days of testing in accordance with NEN 7375 demonstrated that the cumulative amounts of leached components were substantially lower than the reference limit values prescribed by the Regulation on Waste Categories, Testing and Classification.
- Based on the results obtained, it can be concluded that the tested material does not exhibit significant potential for the release of the analyzed contaminants into the environment, indicating favorable environmental stability with respect to leaching behavior.

The obtained diffusion-controlled leaching behavior confirms that the NEN 7375 diffusion test is a suitable tool for assessing the environmental performance of monolithic waste within the Serbian regulatory framework. Its application is consistent with the principles of EU waste legislation and supports waste characterization and landfill acceptance assessment in accordance with Council Decision 2003/33/EC.

To improve the durability of concrete, it is necessary to adjust the water content during mixing. Given the low water absorption of copper slag aggregate, reducing the water content for concrete mixing allows for a lower water–cement ratio to be maintained. This approach not only results in a more compact and durable concrete structure but also contributes to the rational use of natural resources, further promoting the environmental cost-effectiveness of using copper slag aggregate in the construction industry.

It should also be noted that a detailed cost-effectiveness analysis was outside the scope of the present study. The present research was focused on the physical, mechanical, microstructural, and environmental performance of concrete containing coarse copper slag aggregate. However, the economic feasibility of replacing natural river aggregate with copper slag aggregate may depend on material availability, processing requirements, transport distance, and implementation costs. Therefore, future research should include a cost-effectiveness assessment in order to better evaluate the practical applicability of copper slag aggregate in concrete production.

## Figures and Tables

**Table 1:** Physical and mechanical characteristics of aggregates [1].

Test Methods	Standard	Fraction (mm)	Determined Values	
			CCA	RA
Shape index (%)	EN 933-4	8/11	5	
		10/14		16
Resistance to degradation, LA coefficient (%)	SRPS B.B8.045 (ASTM C 131)	grading B	10	27
Resistance to wear, M_DE_ coefficient (%)	EN 1097-1	8/11	4	
		10/14		10
Polished stone value, PSV	EN 1097-8	standardized	43	-
Particle density (Mg/m^3^)	EN 1097-6	8/11	ρ_a_ 3.40	
			ρ_rd_ 3.33	
			ρ_ssd_ 3.36	
		10/14		ρ_a_ 2.66
				ρ_rd_ 2.58
				ρ_ssd_ 2.61
Water absorption, WA_24_ (%)	EN 1097-6	8/11	0.6	
		10/14		1.2
Magnesium sulfate test, MS (%)	EN 1367-2	10/14	7	12

Note: CCA—coarse copper slag aggregate; RA—river aggregate; ρ_a_—apparent particle density; ρ_rd_—oven-dried particle density; ρ_ssd_—saturated and surface-dried particle density.

**Table 2:** Chemical composition of coarse copper slag aggregate (CCA) [1].

Component	Content (%)	Analytical Method
SiO_2_	42.54	G
Al_2_O_3_	3.57	ICP-AES
FeO	22.17	V
Fe_2_O_3_	6.92	R
Fe_3_O_4_	1.23	A-Fe_3_O_4_
CaO	20.29	AAS
MgO	0.51	AAS
SO_3_	1.18	CCA
K_2_O	0.43	AES
K	0.36	R
Na_2_O	0.15	AES
Na	0.11	R
TiO_2_	0.28	ICP-AES
Mn_2_O_3_	0.042	AAS
Cl^−^	<0.01	FOT/SF
Cu-ox	0.96	AAS
P_2_O_5_	0.094	ICP-AES
Cu	0.30	AAS/EG

Explanation: G—gravimetry, V—volumetry, AAS—atomic absorption spectrometry, ICP-AES—inductively coupled plasma atomic emission spectrometry, R—calculation, A-Fe_3_O_4_—magnetite determination, CCA—combustion chemical analysis, AES—atomic emission spectrometry, FOT/SF—photometry/spectrophotometry, AAS/EG—atomic absorption spectrometry.

**Table 3:** Physical and mechanical properties of PC.

Sample	Blaine	Residue R90	Water for Standard Consistency	Initial Setting Time	Flexural Strength 2 Days	Flexural Strength 28 Days	Compressive Strength 2 Days	Compressive Strength 28 Days
UOM	cm^2^/g	%	%	min	MPa	MPa	MPa	MPa
CEM II/A-L 42.5R	4130	0.1	27.4	170	5.3	8.1	29.8	54.1

Note: UOM—unit of measurement; Residue R90—residue on the 90 μm sieve.

**Table 4:** The quantities of aggregate fractions in the concrete mixtures.

Fraction (mm)	Participation (%)
0/4 (I)	40
4/8 (II)	15
8/16 (III)	20
16/32 (IV)	25

**Table 5:** Concrete mix design.

Mixture	Water	RA				CCA		Cement	W/CM
	m_v_	[0/4]	[4/8]	[8/16]	[16/32]	[8/16]	[16/32]	m_c_	ω
	[kg/m^3^]	[kg/m^3^]	[kg/m^3^]	[kg/m^3^]	[kg/m^3^]	[kg/m^3^]	[kg/m^3^]	[kg/m^3^]	[-]
CS0	212	732	274	343	428	-	-	398	0.533
CS1				274	343	94	118		
CS2				172	214	236	294		

Explanation: RA—river aggregate; CCA—coarse copper slag aggregate; W/CM—water-to-cementitious materials ratio.

**Table 6:** Test results of fresh concrete properties.

Mixture	Slump [cm]	Flow [cm]	Bulk Density [kg/m^3^]	Air Content [%]
CS0	20	38.5	2331	1.9
CS1	21	36	2424	1.7
CS2	13	32	2456	1.4

CS0—control mixture; CS1—content of 20% CCA; CS2—content of 50%/100% CCA.

**Table 7:** Petrographic composition of river aggregate and its mixtures with copper slag for concrete.

Group	Type	a	b	c	d	e	f
		Standard	20 III & 20 IV	50 III & 50 IV	50 III & 100 IV	20 III & 50 IV	100 III & 50 IV
igneous	gabbro-diabase	**6.4**	**5.8**	4.8	**3.7**	5.1	4.4
	dacite-andesite	**5.3**	**4.5**	3.4	**1.9**	3.6	2.9
	peridotite	**9.6**	**8.7**	7.3	**6.5**	8.2	5.8
sedimentary	limestone	**3.8**	**3.3**	2.4	**1.7**	2.8	1.8
	sandstone	**10.0**	**8.6**	6.4	**4.3**	7.3	5.0
	chert	**2.9**	**2.6**	2.1	**1.8**	2.4	1.8
	conglomerate	**0.9**	**0.8**	0.7	**0.5**	0.7	0.6
metamorphic	quartzite	**32.9**	**29.9**	25.3	**20.9**	27.2	22.1
	schist	**1.7**	**1.5**	1.1	**0.8**	1.3	0.7
	gneiss	**10.1**	**9.1**	7.5	**6.6**	8.5	6.0
minerals	minerals (Q, F, Fm, M)	**16.5**	**16.5**	16.5	**16.5**	16.5	16.5
slag	copper slag	**0.0**	**9.0**	22.5	**35.0**	16.5	32.5
		**100.0**	**100.0**	100.0	**100.0**	100.0	100.0

Fraction III: 8/16 mm, fraction IV: 16/32 mm; Q—quartz, F—feldspars, Fm—ferro-magnesian minerals, M—mica. Explanation: The bold formatting is used to indicate the three aggregate compositions that were selected for concrete production and further testing from the six compositions initially considered.

**Table 8:** Mechanical properties of hardened concrete containing CCA.

			Compressive Strength [MPa]				Standard Deviation			
	Mixture	Water Penetration Depth [mm]	Curing Period			Bulk Density [kg/m^3^]	Curing Period			Bulk Density [kg/m^3^]
			1 Day	7 Days	28 Days		1 Day	7 Days	28 Days	
CS0	Sample 1	87	16.1	26.2	38.3	2356	16.7 ± 0.5	28.0 ± 2.2	36.3 ± 1.8	2329 ± 24.6
	Sample 2	78	16.8	30.5	34.8	2326				
	Sample 3	85	17.1	27.4	35.7	2307				
CS1	Sample 1	102	17.0	26.4	39.6	2308	17.6 ± 1.0	27.2 ± 0.7	40 ± 1.0	2355 ± 63.2
	Sample 2	85	18.9	27.7	41.6	2427				
	Sample 3	93	16.9	27.5	41.1	2329				
CS2	Sample 1	90	23.2	36.2	40.4	2455	21.8 ± 1.2	35.5 ± 0.6	42.3 ± 1.7	2465 ± 46.0
	Sample 2	112	20.6	34.9	43.6	2516				
	Sample 3	104	21.5	35.2	43.0	2425				

Explanation: Control—control mixture; III—CCA fraction 8/16 mm; IV—CCA fraction 16/32 mm. The results represent individual measurements from three distinct specimens per group, and the standard deviation values are expressed relative to the calculated mean.

**Table 9:** Data on surface area, volume, amount of leaching solution used, and coverage factor.

Sample	Shape of the Sample	Surface, (m^2^)	Fluid Volume, V_f,_ (L)	Coverage Factor
CS0	Half roller: Diameter, R = 0.15 m, height, H = 0.07 m	0.04465	3.5	5.7
CS1, 20% of CS	Half roller: Diameter, R = 0.15 m, height, H = 0.075 m	0.04658	3.5	5.3

**Table 10:** Parameter results obtained during the test.

Sample	Total Mass Loss (g/m^2^)	pH Value	Conductivity	The Weight Loss m_va_ in Stages 1 to 2 (g/m^2^)	The Weight Loss m_vb_ in Stages 2 to 8 (g/m^2^)
CS0	1.41	7.96–10.65	92–197	0.605	1.218
CS1, 20% of CS	3.10	8.25–10.38	92–107	1.047	1.595

**Table 11:** Incremental analysis.

Concentration Parameter	CF 2–7	CF 5–8	CF 4–7	CF 3–6	CF 2–5	CF 1–4	Conclusion
Parameter				Sample CS0			
Sb	1.000	1.000	1.000	1.000	1.000	1.000	n.a
As	1.000	1.000	1.000	1.000	1.000	1.000	n.a
Ba	1.039	1.003	1.030	1.058	1.058	1.056	n.a
Cu	1.000	1.000	1.000	1.000	1.000	1.000	n.a
Hg	0.800	0.800	0.800	0.800	0.800	0.625	n.a
Cd	1.000	1.000	1.000	1.000	1.000	1.000	n.a
Mo	1.000	1.000	1.000	1.000	1.000	1.000	n.a
Ni	1.000	1.000	1.000	1.000	1.000	1.000	n.a
Pb	1.000	1.000	1.000	1.000	1.000	1.000	n.a
Se	1.000	1.000	1.000	1.000	1.000	1.000	n.a
Cr	2.096	3.312	2.644	1.859	1.620	1.250	n.a
Zn	1.203	1.193	1.242	1.344	1.314	1.000	n.a
Ca	2663.09	1999.98	2259.12	2735.00	2784.70	3198.65	F2
F^−^	11.387	8.333	7.254	10.688	11.265	13.266	F2
Cl^−^	39.501	26.046	28.634	48.940	43.236	44.624	F2
SO_4_^2−^	1.892	3.150	1.836	1.762	1.895	1.973	F2
**CS1, 20% of CS**							
Sb	1.000	1.000	1.000	1.000	1.000	1.000	n.a
As	1.000	1.000	1.000	1.000	1.000	1.000	n.a
Ba	2.611	3.333	3.361	3.417	3.417	1.083	n.a
Cu	1.000	1.000	1.000	1.000	1.000	1.000	n.a
Hg	0.800	0.800	0.800	0.800	0.800	0.625	n.a
Cd	1.000	1.000	1.000	1.000	1.000	1.000	n.a
Mo	1.000	1.000	1.000	1.000	1.000	1.000	n.a
Ni	1.000	1.000	1.000	1.000	1.000	1.000	n.a
Pb	1.000	1.000	1.000	1.000	1.000	1.000	n.a
Se	2.375	1.688	2.063	3.063	3.063	2.500	F2
Cr	1.928	3.237	2.360	1.726	1.592	1.200	n.a
Zn	1.000	1.000	1.000	1.000	1.000	1.000	n.a
Ca	2027.37	2198.37	2081.95	2234.34	2149.60	1693.80	F2
F^−^	12.147	11.712	12.087	12.517	12.876	11.315	F2
Cl^−^	35.267	38.107	39.667	41.015	29.456	28.730	F2
SO_4_^2−^	2.430	2.733	2.338	2.172	3.213	3.286	F2

n.a—the leaching mechanism is not applicable for the indicated components. F2—for the indicated components, continue to Step 2.

**Table 12:** Overview of concentration factors, slope, and associated standard deviation in individual increments by components for sample CS0.

Increment a–b	${CF}_{a-b}$	rc	sd_rc_	According to NEN 7375, Point 8.3.2.
Increment 2–7	2663.09	−0.279	31.49	Surface wash-off
Increment 5–8	1999.98	−0.198	13.97	Depletion
Increment 4–7	2259.12	−0.163	17.89	Depletion
Increment 3–6	2735.00	−0.200	17.89	Depletion
Increment 2–5	2784.70	−0.412	19.74	Depletion
Increment 1–4	3198.65	−0.341	24.08	Surface wash-off
Increment 2–7	11.39	−0.013	0.40	Surface wash-off
Increment 5–8	8.33	−0.029	0.22	Depletion
Increment 4–7	7.25	−0.023	1.84	Depletion
Increment 3–6	10.69	−0.016	1.84	Depletion
Increment 2–5	11.27	0.000	1.84	Depletion
Increment 1–4	13.27	0.002	1.85	Surface wash-off
Increment 2–7	39.50	−0.222	6.96	Surface wash-off
Increment 5–8	26.05	−0.223	2.52	Depletion
Increment 4–7	28.63	−0.228	4.24	Depletion
Increment 3–6	48.94	−0.231	4.24	Depletion
Increment 2–5	43.24	−0.222	5.03	Depletion
Increment 1–4	44.62	−0.216	6.48	Surface wash-off
Increment 2–7	1.89	−0.186	8.53	Surface wash-off
Increment 5–8	3.15	−0.204	6.40	Depletion
Increment 4–7	1.84	−0.193	3.86	Depletion
Increment 3–6	1.76	−0.187	3.86	Depletion
Increment 2–5	1.90	−0.179	3.70	Depletion
Increment 1–4	1.97	−0.168	3.99	Surface wash-off

**Table 13:** Overview of concentration factors, slope, and associated standard deviation in individual increments by components for Sample CS1, 20% of CS.

Increment a–b	${CF}_{a-b}$	rc	sd_rc_	According to NEN 7375, Point 8.3.2.
CS1, 20% of CS				
Se				
Increment 2–7	2.38	−0.423	0.03	Surface wash-off
Increment 5–8	1.69	−0.107	0.01	Depletion
Increment 4–7	2.06	−0.170	1.83	Depletion
Increment 3–6	3.06	−0.838	1.83	Depletion
Increment 2–5	3.06	−0.809	1.83	Depletion
Increment 1–4	2.50	−0.390	1.83	Surface wash-off
Ca				
Increment 2–7	2027.37	−0.394	30.03	Surface wash-off
Increment 5–8	2198.37	−0.249	24.17	Depletion
Increment 4–7	2081.95	−0.262	17.32	Depletion
Increment 3–6	2234.34	−0.320	17.32	Depletion
Increment 2–5	2149.60	−0.594	19.91	Depletion
Increment 1–4	1693.80	−0.366	19.80	Surface wash-off
F^−^				
Increment 2–7	12.15	−0.269	0.35	Surface wash-off
Increment 5–8	11.71	−0.182	0.17	Depletion
Increment 4–7	12.09	−0.229	1.86	Depletion
Increment 3–6	12.52	−0.250	1.86	Depletion
Increment 2–5	12.88	−0.349	1.86	Depletion
Increment 1–4	11.31	−0.230	1.86	Surface wash-off
Cl^−^				
Increment 2–7	35.27	−0.206	5.38	Surface wash-off
Increment 5–8	38.11	−0.273	4.66	Depletion
Increment 4–7	39.67	−0.215	6.07	Depletion
Increment 3–6	41.02	−0.209	6.07	Depletion
Increment 2–5	29.46	−0.450	6.55	Depletion
Increment 1–4	28.73	−0.210	6.74	Surface wash-off
SO_4_^2−^				
Increment 2–7	2.43	−0.288	29.55	Surface wash-off
Increment 5–8	2.73	−0.307	30.71	Depletion
Increment 4–7	2.34	−0.173	3.80	Depletion
Increment 3–6	2.17	−0.228	3.80	Depletion
Increment 2–5	3.21	−0.208	6.31	Depletion
Increment 1–4	3.29	−0.250	6.36	Surface wash-off

**Table 14:** Incremental analysis—Step 2 (concentration factor, slope, and standard deviation for increments 5 to 8) for sample CS0 and sample CS1, 20% of CCA.

Increment 5 to 8	CF_5–8_	rc	Criterion 3. rc > 0.8	sd	According to NEN 7375, Point 8.3.3.
CS0					
Ca	1999.98	−0.198	Not satisfied	13.97	The matrix is insoluble
F^−^	8.33	−0.029	Not satisfied	0.22	
Cl^−^	26.05	−0.223	Not satisfied	2.52	
SO_4_^2−^	3.15	−0.204	Not satisfied	6.40	
CS1, 20% of CCA					
Se	1.69	−0.107	Not satisfied	0.01	The matrix is insoluble
Ca	2198.37	−0.249	Not satisfied	24.17	
F^−^	11.71	−0.182	Not satisfied	0.17	
Cl^−^	38.11	−0.273	Not satisfied	4.66	
SO_4_^2−^	2.73	−0.307	Not satisfied	30.71	

**Table 15:** Results of physicochemical and chemical tests of waste (according to the NEN 7375 standard for monolithic waste).

Parameter	Unit	Measurement Uncertainty	Found Value (64th Day of Test)		Reference Value ^(1)^
Sample		%	CS0	CS1, 20% of CCA	
pH value					>6
(20 ± 5 °C)	-	/	7.96–10.65	8.25–10.38	-
Conductivity	(μS/cm)	/	92–197	92–107	0.3
Antimony, Sb	(mg/m^2^ kg dm)	±4.44	0.0002	0.0002	1.3
Arsenic, As	(mg/m^2^ kg dm)	±4.82	0.0006	0.0006	45
Barium, Ba	(mg/m^2^ kg dm)	±4.54	0.0002	0.0006	45
Copper, Cu	(mg/m^2^ kg dm)	±4.76	0.0003	0.0002	0.1
Mercury, Hg	(mg/m^2^ kg dm)	±5.02	0.0001	0.0001	0.2
Cadmium, Cd	(mg/m^2^ kg dm)	±5.36	0.0001	0.0001	7
Molybdenum, Mo	(mg/m^2^ kg dm)	±1.88	0.0002	0.0002	6
Nickel, Ni	(mg/m^2^ kg dm)	±3.78	0.0002	0.0002	6
Lead, Pb	(mg/m^2^ kg dm)	±5.18	0.0006	0.0006	0.4
Selenium, Se	(mg/m^2^ kg dm)	±5.28	0.0001	0.0003	5
Total chromium, Cr_tot_	(mg/m^2^ kg dm)	±4.04	0.0004	0.0004	30
Zinc, Zn	(mg/m^2^ kg dm)	±5.72	0.0002	0.0002	-
Calcium, Ca	(mg/m^2^ kg dm)	±3.64	0.418	0.297	60
F^−^	(mg/m^2^ kg dm)	±2.62	0.006	0.007	10,000
Cl^−^	(mg/m^2^ kg dm)	±5.01	0.093	0.087	10,000

^(1)^ Rulebook on Categories, Testing and Classification of Waste (Official Gazette of the Republic of Serbia No. 56/2010, 93/2019, 39/2021, 65/2024), Annex 8, Part 2. Disposal of non-reactive hazardous [21].

## Data Availability

The original contributions presented in this study are included in the article. Further inquiries can be directed to the corresponding author.
